# Crystal structure of *catena*-poly[bis(formato-κ*O*)bis­[μ_2_-1,1′-(1,4-phenyl­ene)bis­(1*H*-imidazole)-κ^2^ *N* ^3^:*N* ^3′^]cobalt(II)]

**DOI:** 10.1107/S2056989015014255

**Published:** 2015-08-06

**Authors:** Guo-Wang Xu, Ye-Nan Wang, Hong-Xu Xia, Zhong-Long Wang

**Affiliations:** a College of Science, China Three Gorges University, Yichang 443002, People’s Republic of China; b College of Mechanical and Power Engineering, China Three Gorges University, YiChang 443002, People’s Republic of China; c College of Materials and Chemical Engineering, China Three Gorges University, YiChang 443002, People’s Republic of China

**Keywords:** crystal structure, Co complex, 1,4-bis­(1-imidazol­yl)benzene, hydrogen bonding

## Abstract

A red block-shaped crystal of the title compound, [Co(HCOO)_2_(C_12_H_10_N_4_)_2_]_*n*_, was obtained by the reaction of cobalt(II) nitrate hexa­hydrate, formic acid and 1,1′-(1,4-phenyl­ene)bis­(1*H*-imidazole) (bib) mol­ecules. The asymmetric unit consists of one Co^II^ cation, one formate ligand and two halves of a bib ligand. The central Co^II^ cation, located on an inversion centre, is coordinated by two carboxyl­ate O atoms and four N atoms from bib ligands, completing an octa­hedral coordination geometry. The Co^II^ centres are bridged by bib ligands, giving a two-dimensional net. Topologically, taking the Co^II^ atoms as nodes and the bib ligands as linkers, the two-dimensional structure can be simplified as a typical **sql**/Shubnikov tetra­gonal plane network. The structure features C—H⋯O hydrogen-bonding inter­actions between formate and bib ligands, resulting in a three-dimensional supra­molecular network.

## Related literature   

For metal–organic framework structures, see: Yang *et al.* (2011 ▸, 2012 ▸); Guo & Sun (2012 ▸). 
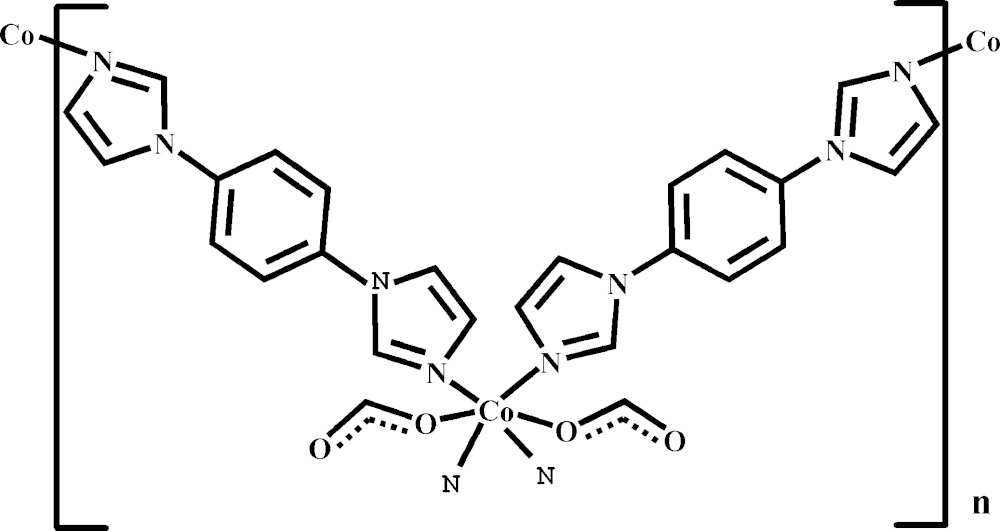


## Experimental   

### Crystal data  

[Co(CHO_2_)_2_(C_12_H_10_N_4_)_2_]
*M* *_r_* = 569.44
Monoclinic, 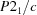

*a* = 7.601 (6) Å
*b* = 11.715 (9) Å
*c* = 13.791 (10) Å
β = 99.017 (9)°
*V* = 1212.8 (16) Å^3^
*Z* = 2
Mo *K*α radiation
μ = 0.76 mm^−1^
*T* = 293 K
0.20 × 0.18 × 0.17 mm

### Data collection  

Bruker SMART 1000 CCD diffractometer
Absorption correction: multi-scan (*SADABS*; Bruker, 2007 ▸) *T*_min_ = 0.732, *T*_max_ = 1
12595 measured reflections
2773 independent reflections
2059 reflections with *I* > 2σ(*I*)
*R*_int_ = 0.092

### Refinement  

*R*[*F* ^2^ > 2σ(*F* ^2^)] = 0.053
*wR*(*F* ^2^) = 0.121
*S* = 1.07
2773 reflections
178 parameters
H-atom parameters constrained
Δρ_max_ = 0.33 e Å^−3^
Δρ_min_ = −0.41 e Å^−3^

### 

Data collection: *SMART* (Bruker, 2007 ▸); cell refinement: *SAINT-Plus* (Bruker, 2007 ▸); data reduction: *SAINT-Plus*; program(s) used to solve structure: *SHELXTL* (Sheldrick, 2008 ▸); program(s) used to refine structure: *SHELXL2014* (Sheldrick, 2015 ▸); molecular graphics: *SHELXTL*; software used to prepare material for publication: *publCIF* (Westrip, 2010 ▸).

## Supplementary Material

Crystal structure: contains datablock(s) I, New_Global_Publ_Block. DOI: 10.1107/S2056989015014255/ff2140sup1.cif

**Fig. 1.**
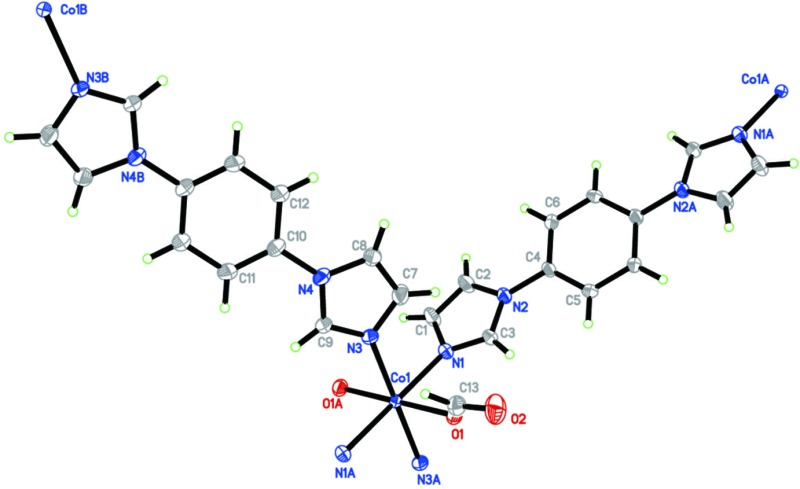
A part of the crystal structure of the title compound with labeling and displacement ellipsoids drawn at the 30% probability level, The H atoms have been removed for clarity. Symmetry codes: (A) -x, 2 - y, 1 - z; (B) 1 - x, 1 - y, -z.

Structure factors: contains datablock(s) I. DOI: 10.1107/S2056989015014255/ff2140Isup2.hkl

x y z x y z . DOI: 10.1107/S2056989015014255/ff2140fig1.tif
A part of the crystal structure of the title compound with labeling and displacement ellipsoids drawn at the 30% probability level, The H atoms have been removed for clarity. Symmetry codes: (A) −*x*, 2 − *y*, 1 − *z*; (B) 1 − *x*, 1 − *y*, −*z*.

CCDC reference: 1415558

Additional supporting information:  crystallographic information; 3D view; checkCIF report

## Figures and Tables

**Table 1:** Hydrogen-bond geometry (, )

*D*H*A*	*D*H	H*A*	*D* *A*	*D*H*A*
C2H2O2^i^	0.93	2.40	3.330(5)	173
C6H6O1^i^	0.93	2.49	3.383(5)	161
C8H8O2^ii^	0.93	2.13	3.038(5)	164

Symmetry codes: (i) 
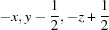
; (ii) 
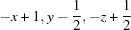
.

## supplementary crystallographic information

### S1. Chemical context

Many fantastic structures of metal-organic frameworks have been reported based on 1,4-bis­(1-imidazolyl)benzene. A lot of the them were constructed by mixed ligands including carboxyl­ate ligand, other N-donor molecule and wolframic acid or molybdic acid, see: Guo *et al.* (2012), Yang *et al.* (2011), Yang *et al.* (2012), Compared with those compounds constructed by mixed organic ligands, coordination polymers established by a single ligand, especially N-donor molecules, are much more useful and easier for understanding the theory of formation of supra­molecules. Accordingly, we selected 1,4-bis­(1-imidazolyl) benzene and cobalt(II), which always shows one or two coordinated ligands, to construct a new supra­molecule and study the structure of the title compound.

### S2. Synthesis and crystallization

The title complex was synthesized by the reaction of 1,4-bis­(1-imidazolyl)benzene ( 10.5 mg, 0.05 mmol) in 8 ml of deionized water with cobalt(II) nitrate hexahydrate ( 29.1 mg, 0.1 mmol) in 20ml of methanol and the mixture was refluxed for 1 hour. To the above mixture, 0.5 ml of formic acid was added and the result fluid was placed in a Teflon-lined, stainless-steel reactor. The reactor was heated to 413 K for 96 hours. It was then cooled to room temperature. Red block crystals were isolated in 69% yield (based on bib ligand).

### S3. Refinement details

All the hydrogen atoms in the molecule were identified from the difference electron density map, further idealized and treated as riding with a distance d(C—H)=0.93Å In all cases Uiso(H)=-1.2Ueq.

### Crystal data

**Table:**

[Co(CHO_2_)_2_(C_12_H_10_N_4_)_2_]	*F*(000) = 586
*M**_r_* = 569.44	*D*_x_ = 1.559 Mg m^−^^3^
Monoclinic, *P*2_1_/*c*	Mo *K*α radiation, λ = 0.71073 Å
*a* = 7.601 (6) Å	Cell parameters from 2599 reflections
*b* = 11.715 (9) Å	θ = 2.3–27.5°
*c* = 13.791 (10) Å	µ = 0.76 mm^−^^1^
β = 99.017 (9)°	*T* = 293 K
*V* = 1212.8 (16) Å^3^	Block, red
*Z* = 2	0.20 × 0.18 × 0.17 mm

### Data collection

**Table:**

Bruker SMART 1000 CCD diffractometer	2059 reflections with *I* > 2σ(*I*)
Detector resolution: 13.6612 pixels mm^-1^	*R*_int_ = 0.092
φ and ω scans	θ_max_ = 27.5°, θ_min_ = 2.7°
Absorption correction: multi-scan (*SADABS*; Bruker, 2007)	*h* = −9→9
*T*_min_ = 0.732, *T*_max_ = 1	*k* = −15→15
12595 measured reflections	*l* = −17→17
2773 independent reflections	

### Refinement

**Table:**

Refinement on *F*^2^	0 restraints
Least-squares matrix: full	Hydrogen site location: inferred from neighbouring sites
*R*[*F*^2^ > 2σ(*F*^2^)] = 0.053	H-atom parameters constrained
*wR*(*F*^2^) = 0.121	*w* = 1/[σ^2^(*F*_o_^2^) + (0.0395*P*)^2^ + 0.7123*P*] where *P* = (*F*_o_^2^ + 2*F*_c_^2^)/3
*S* = 1.07	(Δ/σ)_max_ < 0.001
2773 reflections	Δρ_max_ = 0.33 e Å^−^^3^
178 parameters	Δρ_min_ = −0.41 e Å^−^^3^

### Special details

**Table:**

Geometry. All e.s.d.'s (except the e.s.d. in the dihedral angle between two l.s. planes) are estimated using the full covariance matrix. The cell e.s.d.'s are taken into account individually in the estimation of e.s.d.'s in distances, angles and torsion angles; correlations between e.s.d.'s in cell parameters are only used when they are defined by crystal symmetry. An approximate (isotropic) treatment of cell e.s.d.'s is used for estimating e.s.d.'s involving l.s. planes.

### Fractional atomic coordinates and isotropic or equivalent isotropic displacement parameters (Å^2^)

**Table:**

	*x*	*y*	*z*	*U*_iso_*/*U*_eq_	
Co1	0.0000	1.0000	0.0000	0.02562 (17)	
O1	0.1750 (3)	1.11742 (17)	0.07673 (16)	0.0356 (5)	
N4	0.3551 (3)	0.7072 (2)	0.0552 (2)	0.0357 (6)	
N2	−0.0995 (4)	0.9605 (2)	0.29716 (18)	0.0327 (6)	
N3	0.1856 (4)	0.8626 (2)	0.04773 (19)	0.0340 (6)	
C4	−0.0494 (4)	0.9811 (2)	0.4001 (2)	0.0294 (6)	
N1	−0.1089 (4)	0.9758 (2)	0.13562 (18)	0.0340 (6)	
C6	−0.0753 (4)	0.8960 (3)	0.4668 (2)	0.0330 (7)	
H6	−0.1252	0.8265	0.4446	0.040*	
C11	0.4328 (5)	0.5795 (3)	−0.0706 (3)	0.0452 (9)	
H11	0.3879	0.6324	−0.1184	0.054*	
C5	0.0263 (4)	1.0845 (3)	0.4333 (2)	0.0336 (7)	
H5	0.0442	1.1410	0.3885	0.040*	
C1	−0.2136 (5)	0.8854 (3)	0.1559 (2)	0.0454 (9)	
H1	−0.2780	0.8386	0.1087	0.055*	
C10	0.4314 (4)	0.6030 (3)	0.0271 (2)	0.0361 (7)	
C2	−0.2098 (5)	0.8742 (3)	0.2544 (2)	0.0459 (9)	
H2	−0.2689	0.8198	0.2864	0.055*	
C9	0.2525 (4)	0.7840 (2)	−0.0035 (2)	0.0343 (7)	
H9	0.2326	0.7808	−0.0717	0.041*	
C3	−0.0435 (4)	1.0190 (3)	0.2222 (2)	0.0346 (7)	
H3	0.0318	1.0820	0.2310	0.041*	
C13	0.3415 (5)	1.1113 (3)	0.0969 (2)	0.0422 (8)	
H13	0.3948	1.0518	0.0675	0.051*	
O2	0.4414 (4)	1.1735 (3)	0.1501 (2)	0.0710 (9)	
C12	0.4980 (5)	0.5242 (3)	0.0977 (3)	0.0448 (9)	
H12	0.4969	0.5402	0.1636	0.054*	
C7	0.2476 (5)	0.8367 (3)	0.1452 (3)	0.0527 (10)	
H7	0.2220	0.8785	0.1986	0.063*	
C8	0.3512 (5)	0.7414 (3)	0.1518 (3)	0.0494 (10)	
H8	0.4076	0.7064	0.2088	0.059*	

### Atomic displacement parameters (Å^2^)

**Table:**

	*U*^11^	*U*^22^	*U*^33^	*U*^12^	*U*^13^	*U*^23^
Co1	0.0335 (3)	0.0221 (3)	0.0210 (3)	0.0044 (2)	0.0034 (2)	−0.0006 (2)
O1	0.0393 (13)	0.0303 (11)	0.0360 (12)	0.0005 (10)	0.0022 (10)	−0.0054 (10)
N4	0.0356 (15)	0.0274 (13)	0.0432 (16)	0.0054 (11)	0.0030 (12)	−0.0014 (12)
N2	0.0405 (15)	0.0345 (13)	0.0238 (13)	−0.0026 (11)	0.0080 (11)	−0.0009 (11)
N3	0.0392 (15)	0.0287 (13)	0.0324 (14)	0.0093 (11)	0.0006 (11)	0.0020 (11)
C4	0.0326 (16)	0.0344 (16)	0.0215 (13)	0.0027 (13)	0.0057 (12)	−0.0011 (12)
N1	0.0426 (15)	0.0347 (14)	0.0254 (12)	0.0002 (11)	0.0082 (11)	−0.0004 (11)
C6	0.0431 (18)	0.0277 (15)	0.0283 (15)	−0.0018 (13)	0.0062 (14)	−0.0029 (13)
C11	0.054 (2)	0.0387 (18)	0.043 (2)	0.0143 (17)	0.0071 (17)	0.0073 (16)
C5	0.0444 (19)	0.0303 (16)	0.0268 (15)	−0.0013 (14)	0.0080 (14)	0.0061 (13)
C1	0.052 (2)	0.058 (2)	0.0268 (16)	−0.0157 (18)	0.0059 (15)	−0.0035 (16)
C10	0.0327 (17)	0.0296 (16)	0.0461 (19)	0.0053 (13)	0.0063 (14)	0.0000 (14)
C2	0.058 (2)	0.051 (2)	0.0287 (17)	−0.0178 (18)	0.0064 (16)	0.0003 (16)
C9	0.0393 (18)	0.0290 (15)	0.0343 (17)	0.0070 (14)	0.0046 (14)	0.0017 (13)
C3	0.0447 (19)	0.0346 (17)	0.0258 (15)	0.0006 (14)	0.0100 (13)	0.0012 (13)
C13	0.045 (2)	0.0397 (19)	0.0398 (19)	−0.0021 (16)	0.0017 (16)	−0.0043 (15)
O2	0.0624 (19)	0.078 (2)	0.0664 (19)	−0.0092 (16)	−0.0096 (15)	−0.0266 (17)
C12	0.057 (2)	0.0379 (18)	0.0400 (18)	0.0143 (16)	0.0079 (17)	0.0024 (15)
C7	0.064 (3)	0.047 (2)	0.043 (2)	0.0232 (19)	−0.0058 (18)	−0.0095 (17)
C8	0.063 (3)	0.043 (2)	0.0364 (19)	0.0180 (18)	−0.0110 (17)	−0.0037 (16)

### Geometric parameters (Å, º)

**Table:**

Co1—O1^i^	2.083 (2)	N3—C7	1.387 (4)
Co1—O1	2.083 (2)	C4—C5	1.387 (4)
Co1—N3^i^	2.173 (3)	C4—C6	1.391 (4)
Co1—N3	2.173 (3)	N1—C3	1.320 (4)
Co1—N1	2.179 (3)	N1—C1	1.379 (4)
Co1—N1^i^	2.179 (3)	C6—C5^ii^	1.389 (4)
O1—C13	1.254 (4)	C11—C10	1.377 (5)
N4—C9	1.369 (4)	C11—C12^iii^	1.397 (4)
N4—C8	1.396 (4)	C5—C6^ii^	1.389 (4)
N4—C10	1.431 (4)	C1—C2	1.361 (5)
N2—C3	1.363 (4)	C10—C12	1.379 (4)
N2—C2	1.385 (4)	C13—O2	1.212 (4)
N2—C4	1.432 (4)	C12—C11^iii^	1.397 (4)
N3—C9	1.311 (4)	C7—C8	1.360 (5)
			
O1^i^—Co1—O1	180.0	C9—N3—Co1	130.2 (2)
O1^i^—Co1—N3^i^	90.15 (11)	C7—N3—Co1	124.2 (2)
O1—Co1—N3^i^	89.84 (11)	C5—C4—C6	120.1 (3)
O1^i^—Co1—N3	89.85 (11)	C5—C4—N2	120.4 (3)
O1—Co1—N3	90.16 (11)	C6—C4—N2	119.5 (3)
N3^i^—Co1—N3	180.00 (13)	C3—N1—C1	105.0 (3)
O1^i^—Co1—N1	92.97 (10)	C3—N1—Co1	125.9 (2)
O1—Co1—N1	87.03 (10)	C1—N1—Co1	125.6 (2)
N3^i^—Co1—N1	92.33 (10)	C5^ii^—C6—C4	119.6 (3)
N3—Co1—N1	87.67 (10)	C10—C11—C12^iii^	119.7 (3)
O1^i^—Co1—N1^i^	87.04 (10)	C4—C5—C6^ii^	120.2 (3)
O1—Co1—N1^i^	92.96 (10)	C2—C1—N1	110.7 (3)
N3^i^—Co1—N1^i^	87.67 (10)	C11—C10—C12	119.9 (3)
N3—Co1—N1^i^	92.33 (10)	C11—C10—N4	120.1 (3)
N1—Co1—N1^i^	180.0	C12—C10—N4	119.9 (3)
C13—O1—Co1	128.1 (2)	C1—C2—N2	105.7 (3)
C9—N4—C8	106.2 (3)	N3—C9—N4	112.1 (3)
C9—N4—C10	128.2 (3)	N1—C3—N2	112.0 (3)
C8—N4—C10	125.0 (3)	O2—C13—O1	127.9 (4)
C3—N2—C2	106.5 (3)	C10—C12—C11^iii^	120.3 (3)
C3—N2—C4	127.0 (3)	C8—C7—N3	110.4 (3)
C2—N2—C4	126.5 (3)	C7—C8—N4	105.8 (3)
C9—N3—C7	105.5 (3)		

Symmetry codes: (i) −*x*, −*y*+2, −*z*; (ii) −*x*, −*y*+2, −*z*+1; (iii) −*x*+1, −*y*+1, −*z*.

### Hydrogen-bond geometry (Å, º)

**Table:**

*D*—H···*A*	*D*—H	H···*A*	*D*···*A*	*D*—H···*A*
C2—H2···O2^iv^	0.93	2.40	3.330 (5)	173
C3—H3···O1	0.93	2.57	3.026 (4)	111
C6—H6···O1^iv^	0.93	2.49	3.383 (5)	161
C8—H8···O2^v^	0.93	2.13	3.038 (5)	164

Symmetry codes: (iv) −*x*, *y*−1/2, −*z*+1/2; (v) −*x*+1, *y*−1/2, −*z*+1/2.
