# Supplementary figures and images for: Crystal structure of catena-poly[bis(formato-κO)bis­[μ2-1,1′-(1,4-phenyl­ene)bis­(1H-imidazole)-κ2 N 3:N 3′]cobalt(II)]
Source: Acta Crystallogr E Crystallogr Commun. 2015 Aug 6;71(Pt 9):m156–7. doi: 10.1107/S2056989015014255 (PMC4555424; doi:10.1107/S2056989015014255)

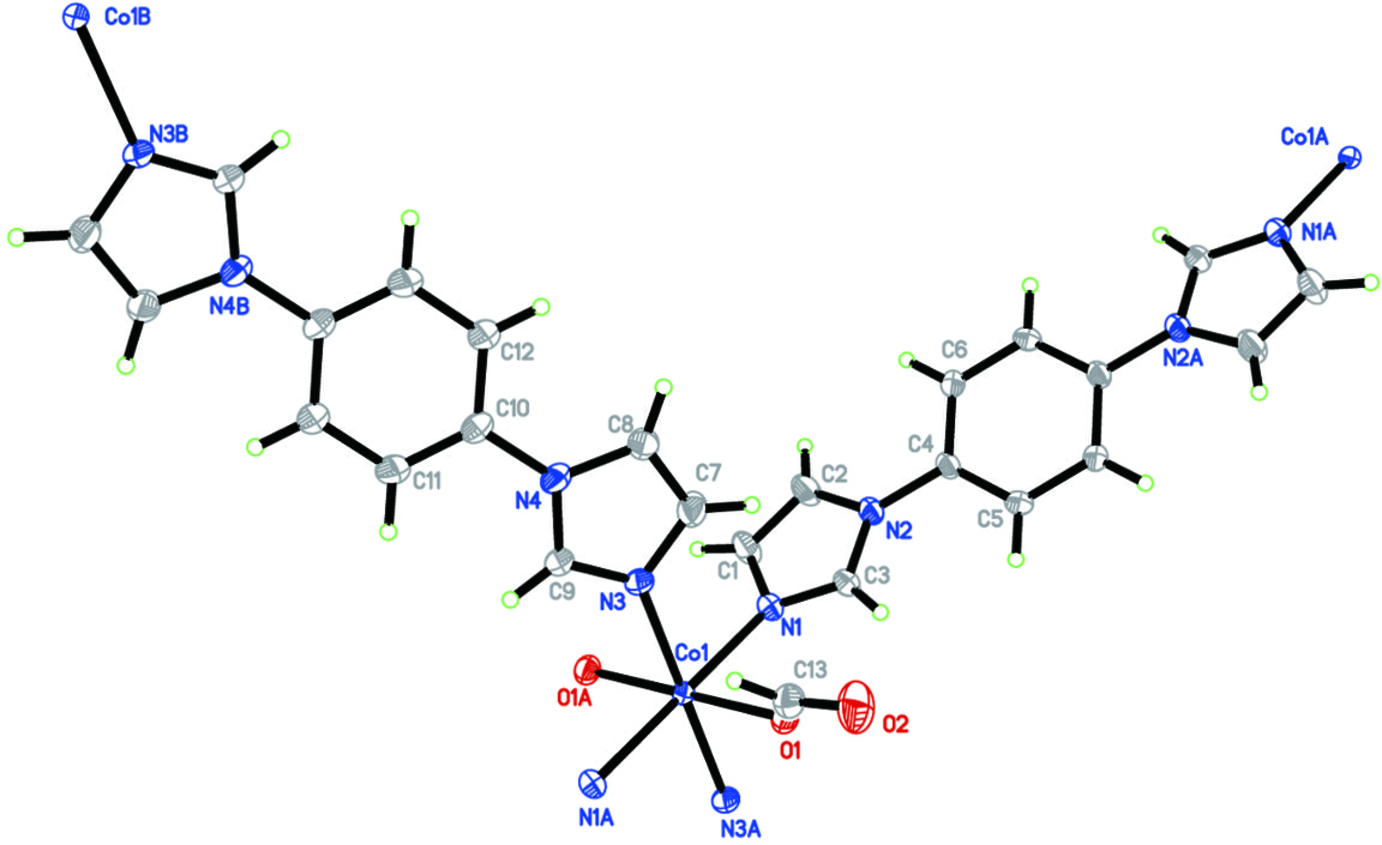

Supplement: Supplementary file 3 [file e-71-0m156-fig1.tif]
